# Resistance training and L-arginine supplementation are determinant in genomic stability, cardiac contractility and muscle mass development in rats

**DOI:** 10.1371/journal.pone.0204858

**Published:** 2018-09-27

**Authors:** Giuseppe Potrick Stefani, Bruna Marmett, Jadson Pereira Alves, Gabriella Berwig Möller, Thiago Gomes Heck, Matias Nunes Frizzo, Marlise Di Domenico, Gabriela Almeida Motta, Pedro Dal Lago, Ramiro Barcos Nunes, Cláudia Ramos Rhoden

**Affiliations:** 1 Laboratory of Experimental Physiology, Federal University of Health Sciences of Porto Alegre (UFCSPA), Porto Alegre, Rio Grande do Sul, Brazil; 2 Graduate Program in Rehabilitation Sciences, Federal University of Health Sciences of Porto Alegre (UFCSPA), Porto Alegre, Rio Grande do Sul, Brazil; 3 Laboratory of Air Pollution, Federal University of Health Sciences of Porto Alegre (UFCSPA), Porto Alegre, Rio Grande do Sul, Brazil; 4 Research Group in Physiology, Post Graduate Program in Comprehensive Health Care, Northwestern Regional University of the State of Rio Grande do Sul (UNIJUÍ), Ijuí, Rio Grande do Sul, Brazil; Max Delbruck Centrum fur Molekulare Medizin Berlin Buch, GERMANY

## Abstract

L-arginine supplementation has been related to increased maximum strength and improvement of hemodynamic parameters in several diseases. The aim of our study was to evaluate the effect of L-arginine supplementation and resistance training on muscle mass, hemodynamic function and DNA damage in healthy rats subjected to a low-arginine concentration diet. Twenty three Wistar rats (290-320g) were divided into 4 groups: Sedentary (SED-Arg, n = 6), Sedentary+Arg (SED+Arg, n = 6), Resistance Training (RT-Arg, n = 5), Resistance Training+Arg (RT+Arg, n = 6). Trained animals performed resistance training protocol in a squat apparatus adapted for rats (4 sets of 10–12 repetitions, 90s of interval, 4x/week, 65–75% of One Maximum Repetition, for 8 weeks). Comet assay was performed to measure DNA damage in leukocytes. The resistance training induced higher muscle mass in trained groups. The L-arginine supplementation increased both gastrocnemius and left ventricle to body mass ratio and increased left ventricle contractility without changing hemodynamic variables. The SED+Arg group showed higher concentration of extracellular heat shock protein 72 (eHSP72) and total testosterone, as well as lower uric acid concentration in blood versus SED-Arg group. The administration of isolated L-arginine supplementation and its association with resistance training promoted less damage in leukocytes DNA. In conclusion, the L-arginine supplementation showed synergistic effect with resistance training regarding leukocyte genomic stability in a low-L-arginine diet scenario.

## Introduction

L-arginine, a semi-essential amino acid, is well known as a precursor for bioactive substance syntheses, such as nitric oxide (NO), growth hormone and creatine [1]. L-arginine is essential for maintaining adequate immune function by improving cell proliferation, increasing antioxidant defenses and enhancing cell protective mechanisms. Across different species, including rodents, pigs and humans, diets with low concentrations of L-arginine lead to decreased circulating levels of this amino acid [2–4].

Since L-arginine demonstrates notable antioxidant properties, such as being a scavenger of reactive oxygen species (ROS) mainly by reducing superoxide and hydroxyl radical oxidative damage [5], a deficient intake of L-arginine may be detrimental to the redox homeostasis of several organs. When the excessive production of ROS overcomes cellular antioxidant capacity, some cellular lesions may occur on different biomolecules; these can be identified, and include lipid peroxidation, protein carbonylation, and DNA strand breaks [6]. DNA damage plays an important role in mutagenesis, carcinogenesis and aging; the accumulation of DNA lesions by ROS has therefore been demonstrated to be a significant risk factor for the development of cardiovascular diseases and cancer [7]. These lesions also alter immune function by impairing of cell proliferation and phagocytosis activity [8,9].

High-intensity or long-term strenuous exercises represents a stress situation that promotes a decrease in indispensable amino acid concentrations [10]; this scenario is accompanied by a release into plasma of substances indicative of muscle damage, mainly due to the production of ROS [11,12]. The use of L-arginine supplements in animal models of exhaustive exercise training has shown increases in antioxidant defense in liver and muscles [13,14], and these effects were accompanied by cytoprotective expressions of 70 kDa heat shock proteins (HSP70) in both cell cytoplasm and nuclei [15]. Exercise-induced HSP70 expression in immune cells, and the release of these proteins from immune cells to the blood (in the extracellular *milieu* named as eHSP70), has also been found to be related to oxidative stress and inflammation in immune cells [16,17].

L-arginine also increases blood flow by endothelium vasodilatation mediated by NO [18]. Similarly, there is evidence that resistance training induces benefits in vascular function mediated by NO modulation and antioxidant effects [19,20]. These benefits to vascular function can also improve cardiac function using both L-arginine supplementation and resistance training [13].

This rationale reinforces the hypothesis that L-arginine supplementation provides increased nutrient support to skeletal muscles and can also improve the removal of metabolites after exercise, making it a promising ergogenic supplement in strength training [21]. Although the parallel benefits of resistance exercise and L-arginine supplementation have been previously studied in terms of their immune system, cardiovascular system and oxidative profiles, the association between resistance training and L-arginine supplementation on these parameters has not yet been investigated. For this study, we hypothesized that resistance exercise and L-arginine supplementation have a synergistic effect on hemodynamic, oxidative, biochemical and biometric improvements.

## Methods

### Animals and experimental design

Male (n = 23), 90-day-old Wistar rats from the Animal Facility of the Federal University of Health Sciences of Porto Alegre (UFCSPA) were kept in semi-metabolic cages (220 x 260 x 310 mm, four animals/cage), under controlled conditions of temperature (24 ± 2 °C), relative humidity (50–60%) and light-dark cycles (light from 7:00 a.m. to 7:00 p.m.). The animals received water and an low-L-arginine diet (22% proteins, 61% sugars, 4% fat, 7% fibers, 1% vitamins, 5% minerals and 0.11% L-arginine ad libitum).

All animals received a low-content L-arginine chow before the study and then were subjected to a resistance training protocol for eight weeks, four times a week (or remained sedentary) and received (or not) L-arginine supplementation (daily by gavage, 500 mg/kg/day). The treatment groups were thus as follows: sedentary animals that received a low-L-arginine diet (Sed-Arg group, n = 6); sedentary animals that received L-arginine supplementation (Sed+Arg group, n = 6); animals subjected to resistance training that received low-L-arginine diet (RT-Arg group, n = 5) and animals subjected to resistance training that received L-arginine supplementation (RT+Arg group, n = 6).

During the 8 weeks of exercise training and L-arginine supplementation, the animals’ body weights were measured. Maximal strength was measured before, during and after the interventions. 24 hours after the last training session, the animals underwent hemodynamic evaluation. Immediately after this procedure, euthanasia was performed for tissue collection. The tissues that were extracted for this study were blood, heart and skeletal muscle (gastrocnemius), which were frozen in liquid nitrogen and kept at—80°C for further biochemical analyses.

### Ethics statement

The handling of animals complied with the *Guide for the Care and Use of Laboratory Animals* published by the National Institutes of Health (NIH publication no. 85–23, revised in 1996) and any other rules applicable to the use of animals for teaching and research, especially resolutions of the National Council for Control of Animal Experimentation. The study was approved by the Ethics Committee on the Use of Animals of the Federal University of Health Sciences of Porto Alegre (UFCSPA) in the process number 114/13.

### Resistance training protocol

The animals in the exercise training groups were subjected to a resistance training protocol, which was performed, after a familiarization period, using a rat adapted squat apparatus for animals. The familiarization period consisted of performing 5 to 10 repetitions at 40–60% of their body weight 3 times a week for 1 week.

The rats were placed in a neoprene vest which held them in lower-limb bipedal position. An electrical stimulus (4–5 mA, 0.3 seconds long, with 3-second intervals between each repetition) was applied to each rat’s tail through a surface electrode, to cause extension and flexion movements of the rat’s lower limbs, thus raising the burden in the squat apparatus. This stimulation was of low intensity and did not cause any injury to the animal’s physical integrity [22]. All training sessions were performed in a dark room.

To determine the maximum load raised in one repetition we used the One Maximum Repetition (1MR) test. From the obtained value, we determined the percentage load for performing the training protocol. In response to the training, the rats gained strength, making it necessary to carry out new tests every 2 weeks to adjust the training load.

The training protocol had a total duration of 8 weeks, with a frequency of 4 times a week; each training session consisted of 4 sets of 12 repetitions with 65–75% of 1MR load with 90 seconds of rest between each series [23]. The training program followed the recommendations of the American Physiological Society (2006).

### L-arginine supplementation

The Groups were supplemented with an L-arginine solution, which was received daily by gavage. The supplement was given in the form of L-arginine powder with a purity of 99.9% (Sigma-Aldrich, SP, Brazil). The supplement dosage used was 500 mg/kg/day diluted in distilled water with total volume of 1.0 mL of solution, since recent studies showed that this concentration of L-arginine was able to significantly increase by approximately 25% the serum levels of nitric oxide [5]. For the animals of the L-arginine-deficient groups, it was performed placebo gavage with the same volume of solution, however, containing only water.

### Evaluation of hemodynamic function

Hemodynamic measurements (blood pressure, heart rate and left ventricular pressure) were recorded 24 hours after the end of the last training session. The animals were anesthetized with ketamine (80 mg/kg, i.p.) + xylazine (12 mg/kg, i.p.). P50 was administered using a polyethylene catheter into the left ventricle via cannulation of the right carotid artery. The catheter was inserted into the left ventricle and its position was determined by observation of a characteristic of the ventricular pressure waveform.

### Recording blood pressure, heart rate and left ventricular pressure

Before placing the PE50 catheter in the ventricle, blood pressure was recorded for 5 minutes through the arterial cannula connection using an electromagnetic transducer (P23 Db, Gould-Statham, USA) connected to a signal preamplifier (Hewlett-Packard 8805C, Puerto Rico, USA). Soon after this measurement, the cannula was positioned in the left ventricle and after 5 minutes of stabilization, ventricular pressure was recorded for 5 minutes. The analog pressure signals were digitalized (CODAS, 1Kz, DTAQ Instruments, USA), allowing for analysis of moment-to-moment blood pressure pulses, with a sampling frequency of 1,000 Hz per channel, to enable assessments of the systolic blood pressure (SBP), diastolic blood pressure (DBP) and mean blood pressure (MBP). The pressure parameters were determined using commercial acquisition software associated with the system. This program allowed for the detection of maximum and minimum pressure curves beat-to-beat, providing the desired variables. MBP was obtained from the integration of pressure values between two consecutive detections of DBP. Heart rate was determined from the systolic interval between two peaks. The left ventricular systolic pressure (LVSP) was determined considering the peak of ventricular contraction.

### Analysis of the contraction and relaxation derivatives of the left ventricle

The analysis of the contraction and relaxation derivatives was based on left ventricular pressure waves recorded in the assessment of hemodynamic function, as performed previously. The analysis was performed using a commercial program associated with the acquisition system. This program allowed for derivation of the wave of left ventricular pressure and the detection of their maximal and minimal curves beat-to-beat, providing the derivatives values of the contraction (+dP/dt_max_) and relaxation (-dP/dt_max_) of the left ventricle.

### Blood and tissue collection

To peform the comet assay and the other biochemical analyzes, it was collected 1.0 ml of whole blood per animal from cannulation through the right carotid artery. After blood collection, the animals were killed by decapitation. At the time of decapitation, the animals were already anesthetized with ketamine (80 mg/kg, i.p.) + xylazine (12 mg/kg, i.p.) for the hemodynamic evaluation. After, it was performed the collection of tissues (heart and gastrocnemius).

### Morphological measures

The morphological measures, namely: the ratio f the mass of the body divided by body mass. This measurement is an indicator of proportionality organs, and the ratio between the left ventricle mass-to-body mass (LV/BM) is also a reliable marker of left ventricular hypertrophy [24]. Also, it was performed the ratio of gastrocnemius mass-to-body mass (G/BM).

### Biochemical and molecular analysis

It was used plasma for determining the concentrations of urea, creatinine, total cholesterol, triglycerides, aspartate aminotransferase (AST), alanine aminotransferase (ALT), uric acid and total testosterone. For obtaining the plasma, the samples were centrifuged at 2,500 rpm for 5 minutes. The analysis were performed by spectrophotometry, using commercial kits of Labtest, following the recommendations of the manufacturer, in a semi-automatic apparatus (Metrolab DR 1600, Wiener). Total testosterone levels were measured by chemiluminescence (Immulite 1000, Siemens, USA). Total protein was measured by refractometry. Extracellular heat shock protein 72 kDa (eHSP72) levels were assessed in plasma with colorimetric ELISA test (HSP70 high sensitivity ELISA Kit—ADI-EKS-715, Enzo Life Sciences). This test recognizes stress-induced HSP70 (HSP72) with negligible reactivity from other HSP70 family members.

### Comet assay alkaline version

The comet assay was performed avoiding direct incidence of light and according to the method of Singh and colleagues (1988) [25]. We used 40 μl of whole blood added to 90 μl of low melting point agarose. After lightly mixed, this material was carefully superimposed on a slide previously covered with a thin agarose gel and covered with a coverslip, and kept in a moist chamber at 4°C for 10 minutes in order to further secure the suspension of blood cells in the gel. Then, the coverslip was carefully removed, and the slide was packaged in a vertical cuvette containing lysis solution for at least 1 hour at 4°C.

The next step consisted in the unfolding of the cells for 5 min in an alkaline buffer (pH > 10.0). Thereafter, followed by the electrophoresis process, where the lysed cells contained in the agarose gel were subjected to a voltage of 25 mV and 300 mA for 15 minutes in a vat containing alkaline buffer solution (pH > 10.0). Then the slide was neutralized, stained with silver nitrate, rinsed and kept at room temperature to dry for later analysis. The slides of each animal were made in duplicate. The analysis was performed by light microscopy of 20X increase, through quantifying the size of the comet’s tail in 100 cells, according to lengths, diameters and dimensions of individual comets. To quantify the damage, it was used as obtained parameters tail moment, Olive tail moment and percentage of tail DNA.

### Quantification of DNA damage

All these parameters were calculated with CASP Labs, Poland) [26]. The percentage of DNA in the tail represents the percentage of DNA stands that have migrated from the comet’s head. The tail moment is the product of the tail length and of the percentage of DNA in the tail. The Olive Tail Moment (OTM) is the product of the distance (relative to the *x*-axis) between the center of gravity of the head with the center of the gravity of the tail of the comet and the % of tail DNA. All formulas are available in supplementary data.

### Statistical analysis

The results are shown as mean ± standard deviation. It was used two-way analysis of variance (ANOVA), followed by the post-hoc of Student-Newman-Keuls, for comparisons among groups. It was considered significant P values < 0.05. Statistical analysis were conducted in SigmaPlot 12.0 software and graphics elaborated in GraphPad Prism 5.0.

## Results

### L-arginine supplementation had no influence in maximum strength but reverts low muscular and ventricular mass

**Fig 1:**
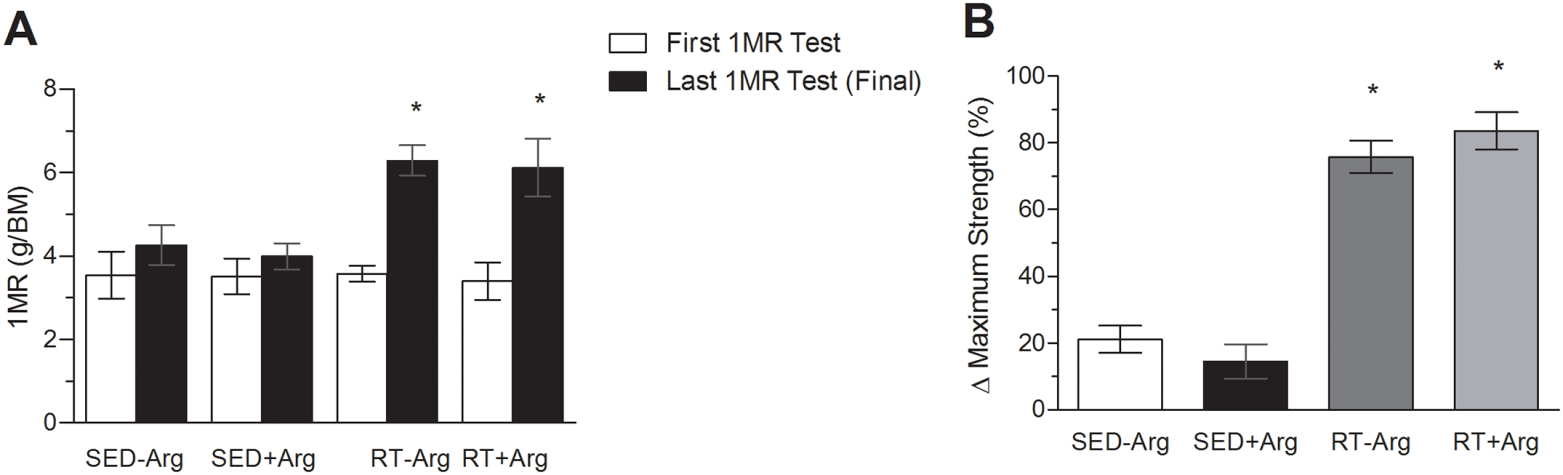
Effects resistance training and L-arginine supplementation on maximum strength gain rats. (A) Basal maximum strength measured by maximal repetition test (1MR) in the first (white bars) and in the last week (black bars) of the study. (B) Maximum strength gain (%) in eight weeks of resistance exercise training. Values in mean ± SD. Two-way ANOVA followed by the post-hoc de Student-Newman-Keuls. n = 5–6 animals/group; * *P*<0,05 vs. SED-Arg and SED+Arg groups.

We evaluated the maximum strength of animals before and after eight weeks of intervention. All groups presented similar maximum strength in the first 1MR test (*P*>0.05) (white bars in Fig 1A). In the last 1MR test, trained groups (RT-Arg and RT+Arg groups) increased maximum strength independent of L-arginine supplementation (*P*<0.05) (black bars in Fig 1A). The increase of maximum strength in trained groups was at least 4 fold higher than sedentary animals (Fig 1B).

At the beginning of the study, all groups started with similar body mass (*P*>0.05) (Table 1). After eight weeks, the SED-Arg, SED+Arg and RT+Arg groups showed an increase in body mass (at least 7.4%, P < 0.05) while RT-Arg group remained with the similar body mass of the first week of the study (Table 1) (P >0.05). The muscle mass index, measured by gastrocnemius to body mass ratio was lower in SED-Arg group than others groups (*P*<0.05). Also, SED+Arg increased LV/BM ratio compared to SED-Arg group (*P*<0.05) (Table 1).

**Table 1:** Morphometric parameters of sedentary, trained and supplemented rats with L-arginine.

	G/BM (mg/g)	LV/BM (mg/g)	Initial Body Mass (g)	Final Body Mass (g)	Δ Body Mass (%)
SED-Arg	4.60 ± 0.16	2.05 ± 0.03	312.67 ± 18.49	336.60 ± 21.88*	+7.40 ± 5.85*
SED+Arg	5.13 ± 0.32**	2.34 ± 0.20**	315.20 ± 14.17	343.00 ± 2.35*	+9.01 ± 5.29*
RT-Arg	5.07 ± 0.55**	2.08 ± 0.31	309.40 ± 16.88	312.40 ± 11.41	+1.08 ± 3.34
RT+Arg	5.32 ± 0.21**	2.05 ± 0.04	313.17 ± 15.28	341.33 ± 4.93*	+10.09 ± 5.43*
*P* Value ANOVA	< 0.05	< 0.05	> 0.05	< 0.05	< 0.05

Values in mean ± SD. Two-way ANOVA followed by the *post-hoc* of Student-Newman-Keuls. n = 5–6 animals/group. LV/BM, Left Ventricle-to-Body Mass Ratio; G/BM, Gastrocnemius-to-Body Mass Ratio.

*, *P*<0.05 compared to RT-Arg group

**; *P*<0.05 compared to SED-Arg group

### L-arginine supplementation and resistance exercise improved hemodynamic parameters

**Fig 2:**
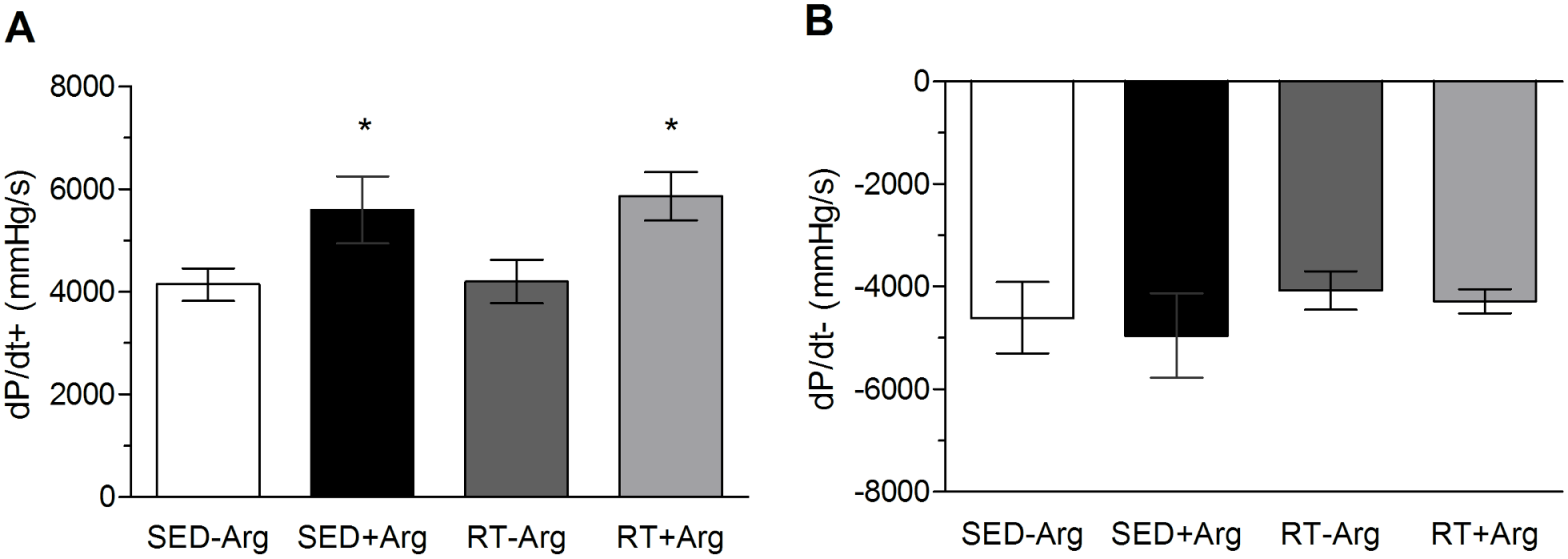
Effects resistance training and L-arginine supplementation on contractility and relaxation of left ventricle. (A) Derivative of contraction (+dP/dt_max_) in mmHg/s. (B) Derivative of relaxation (-dP/dt_max_) in mmHg/s. Values in mean ± SD. Two-way ANOVA followed by the *post-hoc* of Student-Newman-Keuls. n = 5–6 animals/group; * *P*<0,05 vs. SED-Arg.

Hemodynamic data was recorded in rest condition in all animals in the last week of the study. We found no difference among groups in the following hemodynamic parameters: Diastolic, systolic and mean blood pressure, left ventricular systolic pressure and heart rate (*P*>0.05) (Table 2). However, L-arginine supplementation increases left ventricular contractility in sedentary animals and trained rats (Fig 2A) with arginine without modifying diastolic function of left ventricle (Fig 2B).

**Table 2:** Hemodynamic parameters of blood and ventricular pressure of sedentary, trained and supplemented rats with L-arginine.

	DBP (mmHg)	SBP (mmHg)	MBP (mmHg)	LVSP (mmHg)	HR (bpm)
SED-Arg	79.42 ± 14.72	104.42 ± 18.07	91.68 ± 15.06	113.52 ± 21.52	216.01 ± 23.27
SED+Arg	85.72 ± 14.05	107.71 ± 17.81	97.85 ± 15.72	110.32 ± 15.80	215.16 ± 15.51
RT-Arg	75.03 ± 10.77	101.68 ± 17.38	93.25 ± 9.13	100.13 ± 19.19	205.83 ± 19.89
RT+Arg	82.07 ± 17.35	109.69 ± 23.43	90.70 ± 15.63	107.56 ± 21.35	217.18 ± 23.73
*P* Value (ANOVA)	> 0.05	> 0.05	> 0.05	> 0.05	> 0.05

Values in mean ± SD. Two-way ANOVA followed by the *post-hoc* of Student-Newman-Keuls. n = 5–6 animals/group. DBP, Diastolic Blood Pressure; SBP, Systolic Blood Pressure; MBP, Mean Blood Pressure; LVSP, Left Ventricle Systolic Pressure; HR, Heart Rate

### L-arginine supplementation increased eHSP72, testosterone and uric acid levels in sedentary rats

SED+Arg group presented higher levels eHSP72 and total testosterone, and lower levels of uric acid in comparison with SED-Arg group (Table 3). Others biochemical variables were not influenced by L-arginine supplementation or resistance exercise isolated or in association (Table 3).

**Table 3:** Biochemical parameters of rats submitted to resistance training and rats supplemented with L-arginine.

	eHSP72 (ng/mL)	Total Testosterone (ng/mL)	Urea (mg/dL)	Creatinine (mg/dL)	Total Cholesterol (mg/dL)	Triglycerides (mg/dL)	AST (UI/L)	ALT (UI/L)	Uric Acid (mg/dL)
SED-Arg	0.53 ± 0.95	99.24 ± 74.48	33.20 ± 4.97	0.59 ± 0.04	46.60 ± 17.81	45.50 ± 20.73	187.33 ± 113.52	66.50 ± 20.33	1.60 ± 0.73
SED+Arg	5.05 ± 6.09*	398.50 ± 193.37*	35.80 ± 4.27	0.54 ± 0.04	49.00 ± 4.95	43.25 ± 17.86	161.00 ± 59.09	67.67 ± 15.98	0.75 ± 0.45*
RT-Arg	1.24 ± 0.68	200.25 ± 34.64	33.80 ± 3.77	0.56 ± 0.03	41.40 ± 2.70	44.00 ± 4.83	222.20 ± 85.73	65.20 ± 4.27	1.20 ± 0.50
RT+Arg	3.34 ± 2.50	177.20 ± 97.19	34.40 ± 4.28	0.56 ± 0.05	50.80 ± 3.11	48.75 ± 5.38	128.67 ± 50.31	57.33 ± 12.79	0.80 ± 0.34
*P* Value (ANOVA)	< 0.05	< 0.05	> 0.05	> 0.05	> 0.05	> 0.05	> 0.05	> 0.05	< 0.05

Values in mean ± SD. Two-way ANOVA followed by the *post-hoc* of Student-Newman-Keuls. n = 5–6 animals/group. AST, Aspartate Aminotransferase; ALT, Alanine Aminotransferase.

*, *P*<0.05 compared to SED group.

### L-arginine supplementation associated with resistance training decreases DNA damage

**Fig 3:**
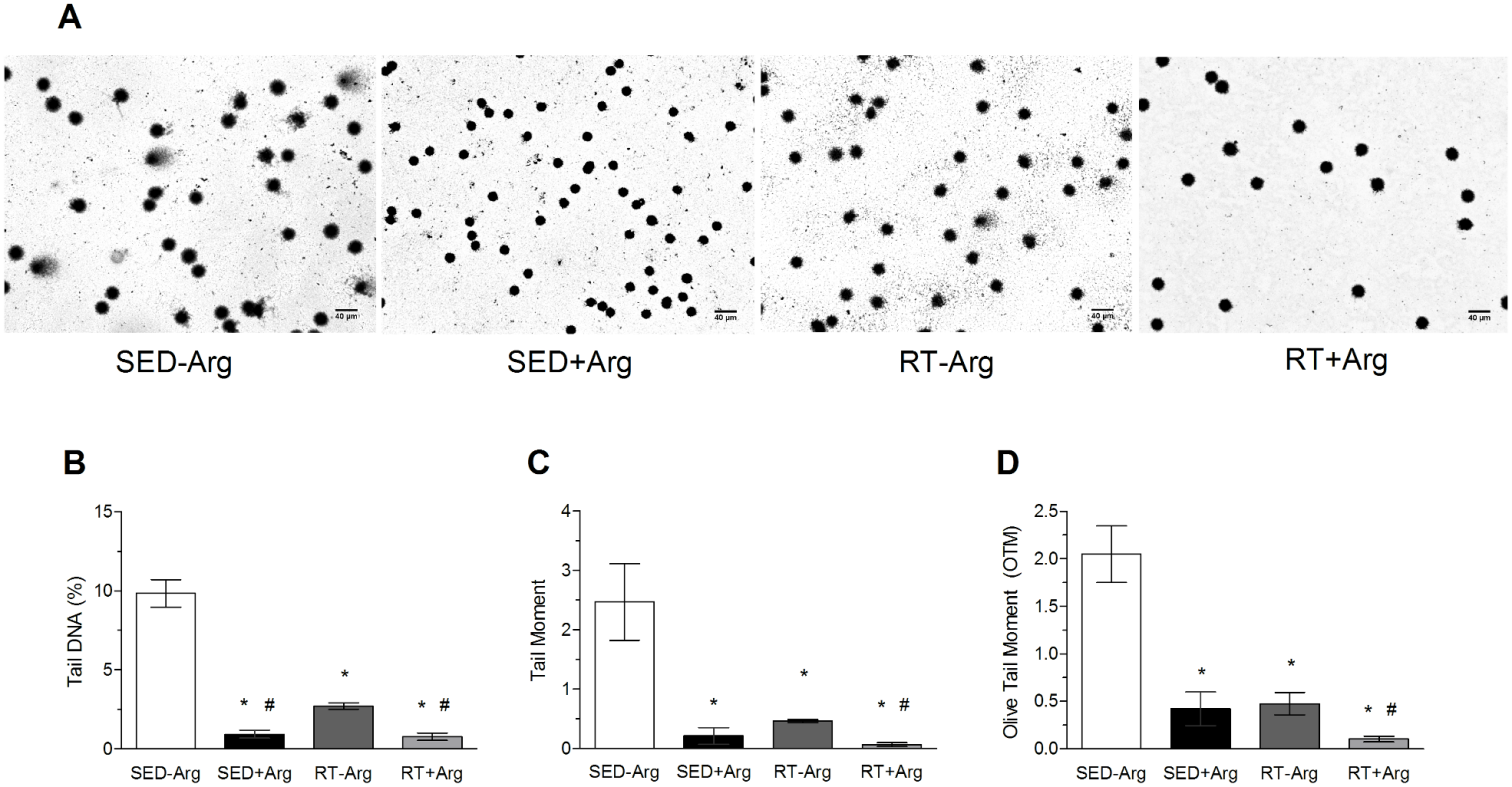
Effects resistance training and L-arginine supplementation on DNA damage of leucocytes. (A) Images captured by optical microscopy of slides containing isolated lymphocytes from whole blood of sedentary and trained rats supplemented with L-arginine. (B) DNA percentage present in the comet’s tail. (C) Tail Moment, value of the product of the tail length and the percentage of DNA in the tail. (D) Olive Tail Moment, value obtained by the difference of center of gravity of the head with the comet’s tail. Values in mean ± SD. Two-way ANOVA followed by the post-hoc of Student-Newman-Keuls. n = 5–6 animals/group; * P<0.05 vs. SED-Arg; # P<0.05 vs. RT-Arg.

In Fig 3A it is possible to observe the presence and frequency of damaged cells (leucocytes) in the experimental groups. When the % tail DNA is evaluated (Fig 3B), the trained groups presented low levels of DNA damage and L-arginine supplementation decreased DNA damage to lowest levels in both SED+Arg and RT+Arg groups. Analyzing tail moment (Fig 3C) and OTM (Fig 3D), we observed a similar decrease in DNA damage induced by L-arginine supplementation and resistance training (compared to SED-Arg group) and also, we found the lowest levels of DNA damage in in RT+Arg group in comparison with all groups (Fig 3C and 3D).

## Discussion

Our study shows novel findings regarding the L-arginine supplementation associated with resistance exercise. The L-arginine supplementation influenced body mass gain and increased left ventricle and gastrocnemius masses (relative to body weight mass). Also, our study supports the hypothesis that L-arginine supplementation promotes hemodynamic functions benefits. Also, for the first time, was demonstrated an effect of L-arginine on eHSP70 and testosterone levels and the decrease of DNA damage in leukocytes. Together, the results of our experimental study reinforce the use of L-arginine as supplement to prevent exercise induced stress under low arginine diet condition.

Resistance exercise promoted a higher strength gain compared to sedentary groups, as expected. However, the trained with supplementation group did not have any further gain of strength. A similar study, with healthy animals, shows that L-arginine supplementation did not influence the performance of exercise in rats [3]. However, an increase in exercise tolerance after L-arginine supplementation is observed only in the presence of pathological conditions accompanied of endothelial dysfunction, such as hypertension and heart failure [27–29]. Thus, since our study represents the condition of healthy subjects, there was no ergogenic effect of L-arginine in trained groups.

It is well know that resistance training is strategy to promote muscle hypertrophy. In our study, supplemented animals and trained groups showed higher gastrocnemius mass-to-body mass ratio compared to SED-Arg group. However, we did not observe gain of body mass of the animals of the RT-Arg group. This fact can be explained by the absence of a possible signaling of L-arginine in the process of muscle protein synthesis, influencing the total body mass gain. The higher muscle mass index observed in trained groups can attributed to the hypertrophy generated by the resistance training while the effect observed in sedentary animals that were supplemented with L-arginine, that showed higher G/BM, can be associated with the increase in the testosterone concentration measured in these rats. These results indicates that L-arginine can influences the testosterone roles in muscle hypertrophy, inducing muscle protein synthesis. This process may be mediated by facilitation of interaction between testosterone and androgen receptor, such as in skeletal muscle and in cardiac muscle [30]. It has been demonstrated that L-arginine can increase mammalian target of rapamycin (mTOR) phosphorilation in skeletal muscle in neonatal piglets [31] and increase muscle gain [32]. Interestingly, it has been already been showed that higher L-arginine availability increased growth of neonatal pigs without increasing plasma insulin or growth hormone [33]. Although in our study, we used adult rats this effect may be similar to those observed in neonatal piglets. However, our data suggests that L-arginine supplementation affects body composition, not being restricted exclusively to animal growth and development. Also, we suppose that RT+Arg group did not showed even higher values of muscle mass than RT-Arg group because the total intake of L-arginine in Low L-arginine chow, even with plus the L-arginine supplementation could not contemplate total L-arginine requirement for muscle growth in resistance trained animals.

Low L-arginine diet showed remarkable differences in left ventricle derivatives of pressure, as well as left ventricle and gastrocnemius mass. A similar investigation of Cremades and colleagues (2004) [34] demonstrated that L-arginine deficient diets influenced the growth of rats. L-arginine deficient diets induce remarkable reduction in muscle and cardiac mass development in lower limb, approximately 20% in relation to the group with standard chow. Our results corroborates with this singular investigation. In this way, our study and previous studies [4,31,35] support the hypothesis that the L-arginine supplementation in situations without physical training, seemed to exert an important function in animal growth and development, mainly in cardiac and skeletal muscle structures. As for the biochemical parameters, no changes were observed among groups, indicating that low L-arginine diet does not seem to affect negatively markers of liver, kidney damage, as well as alter lipid profile.

We found greater eHSP72 plasma concentration only in SED+Arg group. Under amino acid deprivation conditions, heat shock factor 1 (HSF-1), a key transcription factor that activates HSP70 synthesis, presents low activity levels resulting in low HSP70 expression inside the mammalian cells [36]. In this way, L-arginine supplementation increased intracellularly HSP70 levels in the same profile as observed by L-glutamine supplementation [15], a well know enhancer of HSF-1 activity and HSP70 expression. Also, L-arginine consumption induces an increase in eHSP70 levels in serum of rats after acute exercise [15,37,38]. Generally, the release of eHSP72 from cells and tissues to bloodstream denotes a cell signaling, and usually is strongly related to danger signals from immune cells to whole body [17,39,40]. In this way, since intracellularly HSP70 has chaperone action, the eHSP72 can be considered a chaperokine in the circulation, being a cytokine-like signal [41]. Thus, L-arginine supplementation may modify leukocytes response by modulation in eHSP72 levels, which modulates both monocyte and lymphocyte immune activities (phagocytosis and proliferation rates respectively) mainly under exercise situation [17,40].

The higher left ventricle contractility observed in supplemented groups, as demonstrated by the derivative of contractility in left ventricle (+dP/dt_max_), seems to be related to the greater ratio of left ventricular mass-to-body mass. The additional intake of L-arginine seems to have directly influenced by eHSP72 plasma concentration, which was greater in the group that did not performed resistance training. Evidences suggest that cardiomyocyte intracellular HSP70 appears to be associated with the management of myocardial Ca^2+^. The intracellular HSP70 is capable of modulating by increasing the activity of sarcoplasmatic reticulum Ca^2+^-ATPase, as well as augmenting the phosphorylation of ryanodine receptor in cardiomyocytes [42]. Therefore, regulating Ca^2+^ handling factors could increase heart contractility. In addition to this mechanism has the role of HSP70 in the process of cardiac hypertrophy. The same group of sedentary animals showed higher concentration of testosterone, which reinforces our findings that possibly exists a relationship between higher eHSP72 plasma levels and cardiac hypertrophy. Although remains unclear which tissues could release HSP72 to plasma, many cell surface receptors for eHSP70 were described and also, have been suggested that many tissues can uptake and internalize circulating eHSP70 [43,44].

Our study demonstrates that diets with low concentration of L-arginine could exert major function in genomic stability. It is possible that L-arginine supplementation plays an important role on leukocyte replication. In addition, L-arginine potentiates HSP70 expression in many cells and tissues, and overexpression of such proteins is associated to increase nuclear location of HSP70, including conditions related to acute and chronic exercise efforts [15,17]. In other hand, low L-arginine diets may compromise approximately 90% of sperm density and number, as well as stimulates proliferation in intestinal cells [4,32]. It has been demonstrated protective effects of L-arginine administration by partially protecting liver damage in acute cholestasis [45]. In our study the analysis DNA damage showed higher percentage of DNA damage in untrained group (~10%), while leukocytes from resistance trained rats presented less than 3% of DNA damage. It has been demonstrated that the NO in blood could act as antioxidant, inhibiting free radical generation from rat peripheral polymorphonuclear leukocytes [46]. Thus, the lower DNA damage in leukocytes from resistance trained rats that received L-arginine can be attributed to lower pro-oxidant profile in this group. In the same way, resistance training has generated protection in DNA damage possibly due to more efficient mechanisms of nuclear DNA repair [47], but its association with L-arginine supplementation resulted in an even more powerful protection, since dietary factors appears to be crucial in genomic stability [48]. Also, L-arginine is able to down-regulate the enzymatic activity of xanthine oxidase (XO) in different tissues, such as heart, skeletal muscle, liver and lung, when submitted to exhaustive exercise [14]. The protective role of NO remains in preventing the formation of superoxide by XO reaction. In XO biochemical reactions, uric acid is formed as by-product, and it is considered a biomarker for chronic heart failure patients monitoring. In our study we observed lower concentration of uric acid in the SED+Arg group and this finding reinforces the antioxidant effect observed in the dosage supplemented, assuming it is protecting a diet with low concentration of L-arginine.

Shan and colleagues (2013) [5] showed that L-arginine levels were reduced when an increase of inducible nitric oxide synthase (iNOS) activity, and also demonstrated that the supplementaton of L-arginine was able to exert antioxidant activity through NO pathway. In the same study it was shown that the supplementation with the same dosage used in our study (500 mg/kg/day) was able to increase the serum bioavailability of L-arginine in sedentary and trained animals. This increase was possibly caused by singnaling higher expression of iNOS, which would justify the protection in sedentary and supplemented groups. It is noteworthy that such work used in endurance training model. This exercise protocol has distincts physiological and biochemical features of the exercise training protocol that we used, however, both works showed antioxidant effect of L-arginine supplementation, even in different biomarkers of oxidative stress. To our knowledge, we demonstrated for the first time the protective effect of isolated supplementation of L-arginine or associated with resistance training.

This study has few limitations, for example, did not evaluate the concentration of nitrates / nitrites that could better elucidate the role of L-arginine as an antioxidant through a plasma biomarker. As a future perspective to better understand this scenario, the measurement of intracellular HSP70 in the heart and gastrocnemius could justify a possible release of HSP70 to plasma and tissue, which could be contributing more significantly to this signaling in muscle hypertrophy.

## Conclusion

In summary, our findings show that L-arginine supplementation and resistance training promotes benefits regarding biometric, cardiovascular and hormonal aspects accompanied by altered levels of cell stress and DNA damage markers. Together, the results of our study reinforce the use of L-arginine as a supplement to prevent exercise-induced stress under low-arginine diet condition. Studies on L-arginine supplementation may lead to new findings on the effects of this amino acid on different physiological systems and different health conditions. Therefore, further studies are encouraged in order to deepen the knowledge of the effects of L-arginine.

## Supporting information

S1 Table Data of biochemical analyses, hemodynamic parameters and strength gain. (XLSX)
